# New Insights into the Larvicidal Activity of *Leptolegnia chapmanii* Against *Aedes aegypti*: In Vitro and In Vivo Studies

**DOI:** 10.3390/pathogens15060609

**Published:** 2026-06-08

**Authors:** Alaine M. L. Catão, Dulcimê Gonçalves Dorta, Walquíria Arruda, Cristian Montalva, Christian Luz

**Affiliations:** 1 Laboratório de Patologia de Invertebrados, Instituto de Patologia Tropical e Saúde Pública (IPTSP), Universidade Federal de Goiás (UFG), Goiânia 74605-050, GO, Brazil; gondulce@gmail.com (D.G.D.); wolf@ufg.br (C.L.); 2 Facultad de Ciencias, Universidad San Sebastián, Valdivia 5110693, Chile; 3 Instituto de Ciências Biológicas, Departamento de Histologia, Embriologia e Biologia Celular, Universidade Federal de Goiás (UFG), Goiânia 74690-900, GO, Brazil; walqui@ufg.br; 4 Laboratorio de Salud de Bosques, Instituto de Conservación, Biodiversidad y Territorio, Facultad de Ciencias Forestales y Recursos Naturales, Universidad Austral de Chile, Valdivia 5110566, Chile; cristian.montalva@uach.cl

**Keywords:** mosquito, oomycete, cyst germination, histopathology, zoosporangium, biological control

## Abstract

*Leptolegnia chapmanii* is an oomycete pathogen of mosquito larvae. We investigated whether nutritional factors promoting cyst germination in vitro are associated with early infection events and instar-specific susceptibility in *Aedes aegypti*. Cysts of the Brazilian isolate ARSEF 12829 germinated rapidly in soybean seed extract, sunflower seed extract and minimal medium supplemented with yeast extract, whereas basal minimal medium did not promote germination. In sunflower seed extract, germination increased significantly with incubation time; in minimal medium, germination at 24 h was much higher with ≥0.2% yeast extract than with 0.1%. In third-instar larvae, a few cysts attached to the cuticle during the first 30–60 min, with no external germ tubes observed. At 3 h, melanized hyphal structures were detected in the midgut, and histological sections showed germinated and ungerminated cysts in the endoperitrophic space, with hyphae crossing the peritrophic matrix and midgut epithelium toward the hemocoel. Mortality increased with cyst concentration and exposure time and decreased with larval instar. At 3.3 × 10^3^ cysts/mL, final mortality reached 100% in L1–L3 and 91.2% in L4 larvae. These results link rapid cyst germination with early midgut invasion and high larvicidal activity.

## 1. Introduction

*Aedes aegypti* is the principal urban vector of dengue, Zika and chikungunya viruses in tropical and subtropical regions and can also transmit yellow fever virus in urban environments [1,2,3]. In Brazil, the 2024 dengue epidemic demonstrated the continuing difficulty of suppressing this mosquito in densely populated urban environments [4]. Control still largely relies on source reduction and insecticide applications, but operational limitations, behavioral plasticity and widespread resistance to commonly used insecticides have progressively reduced the effectiveness of these measures and reinforced the need for complementary non-chemical tools within integrated vector management [5,6,7,8,9].

*Leptolegnia chapmanii* is an aquatic mosquito-pathogenic oomycete reported from the United States of America and from multiple localities in Argentina and Brazil, and it is pathogenic to larvae of species of the genera *Aedes*, *Anopheles* and *Culex* [10,11,12,13,14,15]. Experimental studies have shown strong larvicidal activity, persistence in containers for several days and limited effects on tested non-target aquatic invertebrates, suggesting its potential as a biological control agent [11,13,15,16,17].

The infection cycle of *L. chapmanii* in mosquito larvae comprises zoosporangial formation on cadavers, release of motile zoospores into water, encystment into cysts, cyst germination, invasion of host tissues and renewed sporulation on dead larvae [13,18]. Classic microscopic studies showed that infection may start either after encysted secondary zoospores attach to the cuticle or after ingestion of zoospore cysts (encysted zoospores) that germinate in the larval midgut [18,19]. Ultrastructural observations further showed that hyphae penetrate the peritrophic matrix and the gut epithelium to reach the hemocoel [18,20]. However, our current understanding of these early infection events still largely relies on studies conducted decades ago, whereas more recent research on *L. chapmanii* has mainly focused on virulence, persistence or compatibility with other larval control agents rather than on linking cyst performance to the earliest host infection processes [16,17,21,22].

In practical terms, culture media that support the rapid production of infective propagules are relevant because propagule quality may influence the initiation of infection. Low-cost seed-based media have been used for mosquito-pathogenic oomycetes, including sunflower-based media for *L. chapmanii* and *Lagenidium giganteum* and soybean-based media for *L. giganteum*, with marked effects on zoospore production and larvicidal activity [23,24,25]. These media were selected because sunflower seed extract has been proposed as an inexpensive substrate for *L. chapmanii* development and zoospore production, whereas soybean seed extract represents another readily available plant-based substrate with nutritional components, including lipids and sterol-related compounds, that may favor infective-stage development in oomycetes [24,25,26]. More generally, studies on sterol metabolism in oomycetes support the idea that plant-derived lipids may influence the development of infective stages [26]. In the host, the larval gut is compartmentalized by the peritrophic matrix, an acellular semipermeable barrier that protects the midgut epithelium and mediates interactions with ingested microorganisms [27,28,29]. In *L. chapmanii*, ingested cysts germinate in the midgut, and the resulting hyphae cross the peritrophic matrix and epithelium to reach the hemocoel [18,20]. Therefore, evaluating whether the in vitro germination response is associated with early invasion and larval-instar susceptibility is relevant both for pathogenesis and for optimizing propagule production.

Here, we investigated the Brazilian *L. chapmanii* isolate ARSEF 12829 by combining in vitro cyst germination assays with in vivo infection experiments, whole-mount observations and histopathology analysis of *A. aegypti* larvae. ARSEF 12829 was selected because it is a Brazilian isolate that was previously obtained from *A. aegypti* larvae, morphologically and molecularly characterized, deposited in ARSEF, and associated with rapid larvicidal activity in the first report of *L. chapmanii* from central Brazil [12]. Specifically, we evaluated whether nutritional conditions that promote germination are associated with rapid infection, which entry route predominates during the first hours after exposure, and how susceptibility changes during the four larval instars.

## 2. Materials and Methods

### 2.1. Origin, Rearing of Aedes aegypti and Preparation of Larvae

The *A. aegypti* colony originated from larvae collected in Goiânia, Brazil, in 2012 and was maintained under laboratory conditions within a controlled environment at 25 ± 5 °C, 75 ± 5% relative humidity (RH) and with a natural photoperiod [30]. Adult females were fed following the protocol described by Lima et al. [31], a technique approved by the Ethics Committee for the Use of Animals at the Universidade Federal de Goiás (CEUA 062/16).

Oviposition and egg handling followed the methods of Luz et al. [32] and Rocha et al. [30]. To hatch eggs, filter papers bearing eggs were transferred to plastic bowls containing 500 mL of tap water and held at ambient temperature. First-instar larvae (L1) were used 24 h after hatching. To obtain later instars (L2–L4), larvae were reared with small amounts of ground commercial cat food pellets (Bom Preço^®^, Salto de Pirapora, Brazil) until they reached the required instar. Food was withheld for 24 h before each assay so that larvae were tested after a fasting period with minimal gut contents.

### 2.2. Origin, Culture of Leptolegnia chapmanii and Preparation of Cysts

*L. chapmanii* ARSEF 12829, a Brazilian isolate previously reported by Montalva et al. [12] and deposited in the USDA-ARS Collection of Entomopathogenic Fungal Cultures (ARSEF; Ithaca, NY, USA), was used in this study. To maintain the culture, the pathogen was grown on peptone-yeast extract-glucose (PYG) medium in 90 × 15 mm Petri dishes for 7 d at 25 ± 1 °C, 75 ± 5% relative humidity and with a 12 h photophase [23].

For cyst production, a 1 cm^3^ block of 7-day-old PYG culture was submerged in a 50 mL plastic cup containing 25 mL of sterile distilled water. After 48 h at 25 ± 1 °C, the block was removed, and the cysts (zoospores released from the mycelium and subsequently encysted) were manually resuspended in the water. Cyst density was determined with a hemocytometer, and suspensions were adjusted to 3.3 × 10^1^, 1.0 × 10^2^, 3.3 × 10^2^, 1.0 × 10^3^, and 3.3 × 10^3^ cysts/mL. Cyst suspensions were routinely prepared 2 h before the in vitro and in vivo experiments.

### 2.3. Preparation of Liquid Media for In Vitro Germination Assays

Cyst germination was evaluated in three liquid media: soybean seed extract (SSE), sunflower seed extract (SFE), and minimal medium supplemented with yeast extract (MMY). SSE was prepared directly for each final concentration by crushing soybean seeds in distilled water at dosages of 0.1, 0.2, or 0.5% (*w*/*v*), which correspond to 1, 2, or 5 g of soybean seeds per liter of distilled water, respectively. SFE was prepared in the same way, using 1, 2, or 5 g of sunflower seeds per liter of distilled water. In all cases, the extracts were filtered, autoclaved, centrifuged, and autoclaved again at 120 °C for 20 min.

The MMY contained 30 g sucrose, 0.01 g Na_2_CO_3_, 0.01 g FeSO_4_·7H_2_O, 0.5 g KCl, and 0.5 g MgSO_4_·7H_2_O in distilled water to a final volume of 1000 mL; yeast extract was added at dosages of 0.1, 0.2 and 0.5% (*w*/*v*) to generate the MMY treatments. MMY without yeast extract served as the negative control. PYG served as positive control. The liquid preparations were autoclaved once at 120 °C for 20 min and stored at 5 °C until use.

### 2.4. Evaluation of Cyst Germination In Vitro

A 0.5 mL aliquot of cyst suspension (1.0 × 10^4^ cysts/mL) was inoculated into 1.5 mL of each liquid medium in 24-well culture plates (15.4 × 5 mm). One plate was prepared for each medium, with one well assigned to each incubation time (2, 4, 8, 12, and 24 h). Plates were incubated at 25 ± 1 °C under a 12 h photophase. At each evaluation time, two drops of lactophenol cotton blue [33] were added to the corresponding well with a plastic pipette. Germination was examined at 400× magnification with a phase-contrast compound microscope (BX41, Olympus, Tokyo, Japan). A total of one hundred cysts were counted in five different microscopic fields per well. Cysts were considered germinated when the germ tube was longer than the cyst diameter. Three independent repetitions were carried out.

### 2.5. Evaluation of Larvicidal Activity

Twenty *A. aegypti* larvae of the same instar (L1, L2, L3 and L4) were exposed to 25 mL of cyst suspension in 50 mL plastic cups and incubated at 25 ± 1 °C under a 12 h photophase for up to 90 h. For each instar, the tested concentrations were 3.3 × 10^1^, 1.0 × 10^2^, 3.3 × 10^2^, 1.0 × 10^3^ and 3.3 × 10^3^ cysts/mL. Control groups of the same instar were maintained in 25 mL of sterile water under identical conditions. Cups were sealed with polyethylene film (Boreda^®^, Contagem, Brazil) to minimize evaporation. Four independent repetitions were carried out for each treatment.

Larval condition was assessed after 1, 2, 3, 4, 5, 6, 7, 8, 12, 24, 36, 48, 72 and 90 h of exposure. At each assessment, the wall of the cup was gently tapped with a plastic pipette, and each larva was then touched lightly with the pipette tip. Larvae showing normal coordinated swimming were classified as alive; larvae showing only weak or uncoordinated movement after stimulation were classified as moribund; and larvae showing no response to either stimulus were classified as dead. Dead larvae were removed, blotted briefly on sterile filter paper, mounted in a drop of sterile water on a microscope slide, and examined immediately under a phase-contrast microscope (DM2500, Leica Microsystems, Wetzlar, Germany) for hyphae and/or zoosporangia on the cuticle or within the body. Cadavers without visible structures were then transferred to a moist chamber (closed container with moistened filter paper, 25 ± 1 °C) and reexamined after 24 h for further development of hyphae and zoosporangia.

### 2.6. Evaluation of In Vivo Infection Process

Twenty-five L3 larvae were placed in a 50 mL plastic cup containing 25 mL of a cyst suspension (1.0 × 10^3^ cysts/mL) and maintained under continuous exposure at 25 ± 1 °C under a 12 h photophase. Control cups containing 25 L3 larvae in sterile distilled water were maintained under the same conditions. Larvae were not fed during the assay. After 30 min and 1, 2, 4, 6, 8, 10, 12, 24, and 48 h of exposure, two individuals were removed from each cup and classified as alive, moribund, or dead according to the criteria defined above. Each specimen was placed on a microscope slide, stained with two drops of lactophenol cotton blue [33], and gently compressed under a coverslip. Cyst germination, hyphal development and zoosporangial formation were examined at 400× magnification with a phase-contrast microscope (DM2500, Leica Microsystems, Wetzlar, Germany). The experiment was repeated three times (n = six larvae per treatment and exposure time).

### 2.7. Histopathology

For histopathological analyses, 20 L3 larvae were exposed to 1.0 × 10^3^ cysts/mL and sampled within the first 3 h of exposure in order to examine early tissue alterations associated with infection. Moribund individuals showing reduced locomotion and cuticular darkening were removed and fixed in 4% paraformaldehyde in 0.1 M sodium phosphate buffer (pH 7.2) for 24 h at 4 °C. This selection was intended to obtain sections representative of ongoing infection rather than of unaffected specimens or advanced postmortem degradation. Samples were then rinsed three times in the same buffer for 15 min, dehydrated through an ethanol series (30, 50, 70, 80, 90 and twice in 100%; 10 min each) and embedded in historesin (Leica HistoResin Embedding Kit^®^, Wetzlar, Germany) for 20 h at 4 °C [34]. Polymerization was carried out overnight at room temperature. Longitudinal sections (4 µm) were obtained with a semi-motorized rotary microtome (Leica Biosystems RM2245^®^, Nussloch, Germany). Sections were stained with hematoxylin–eosin (HE) or periodic acid–Schiff (PAS), examined and photodocumented with a light microscope coupled to the Leica Application Suite EZ imaging system (Leica Microsystems, Wetzlar, Germany).

### 2.8. Analysis of Data

Mortality and germination percentages were analyzed as untransformed values. Before ANOVA, residual normality was assessed using normal probability plots and the Shapiro–Wilk test, and homogeneity of variances was evaluated using Levene’s test. Data were analyzed by ANOVA followed by the Student–Newman–Keuls multiple range test for comparison of means. Means were considered significantly different if *p* < 0.05. Lethal times (LT_50_ and LT_90_), lethal concentrations (LC_50_ and LC_90_), and their 95% confidence intervals were estimated separately by probit analysis for dependent and independent data, respectively (Statistica 7.1; StatSoft, Tulsa, OK, USA) [35,36]. Graphs were generated with SigmaPlot 12.0 (Systat Software Inc., San Jose, CA, USA).

## 3. Results

### 3.1. In Vitro Germination in Seed Extracts and Minimal Medium with Yeast Extract

**Figure 1:**
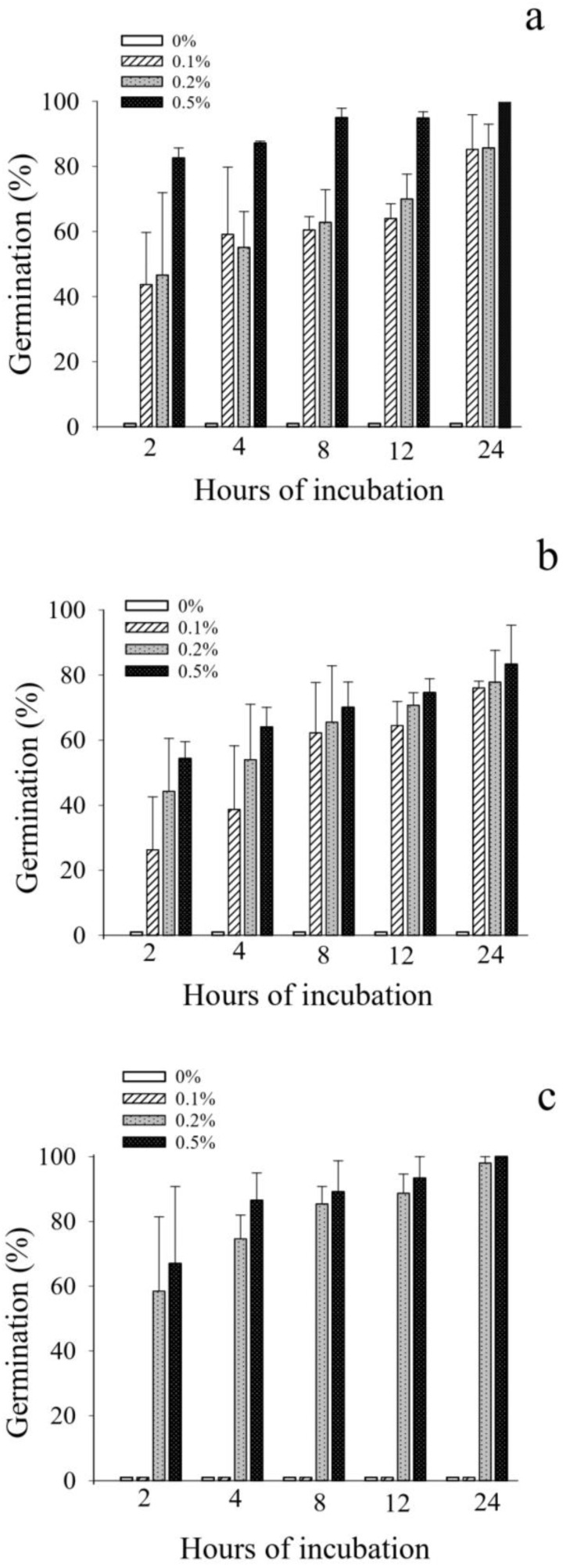
Relative germination (mean ± SE) of *Leptolegnia chapmanii* ARSEF 12829 cysts in soybean seed extract (SSE, (**a**)), sunflower seed extract (SFE, (**b**)), and minimal medium supplemented with yeast extract (MMY, (**c**)) at 0% (control), 0.1%, 0.2%, and 0.5% (*w*/*v*), which were incubated at 25 ± 1 °C under a 12 h photophase for up to 24 h. In the 0% treatment, germination was absent or negligible; therefore, control bars are barely visible because they overlap with the x-axis. Statistical differences among concentrations within each incubation time are described in the text.

Cyst germination was observed in soybean seed extract (SSE) and sunflower seed extract (SFE) at 2 h at all tested concentrations (Figure 1a,b). In SSE, maximum germination reached 100% at 0.5% SSE after 24 h. In SFE, germination was 26.3 ± 16.3% at 0.1% SFE after 2 h and 83.0 ± 12.1% at 0.5% after 24 h. In minimal medium supplemented with yeast extract (MMY), germination remained near zero at 0.1% yeast extract throughout the assay, whereas the values at 0.2% and 0.5% reached ≥98.3 ± 1.7% by 24 h (Figure 1c).

A significant effect of incubation time was detected in SFE (F_4,40_ = 5.04, *p* = 0.002), but not in SSE (F_4,40_ = 2.48, *p* = 0.055) or MMY (F_4,40_ = 2.53, *p* = 0.06). At 24 h, germination did not differ significantly among SSE concentrations (F_2,6_ = 0.92, *p* = 0.45) or among SFE concentrations (F_2,6_ = 0.18, *p* = 0.84), whereas it differed among MMY treatments (F_2,6_ = 2806, *p* < 0.001). Although the mean germination values in SSE and SFE tended to be higher at the highest concentrations, the differences at 24 h were not statistically supported. This lack of statistical support may reflect variability among independent replicates and the limited number of replicate assays.

**Figure 2:**
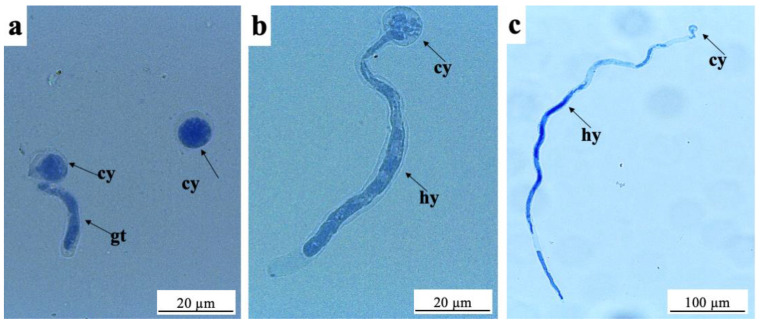
Representative morphology of *Leptolegnia chapmanii* ARSEF 12829 cyst germination in minimal medium supplemented with 0.2% yeast extract, which is used to illustrate the germination criterion applied in the in vitro assays. Specimens were incubated for up to 24 h at 25 ± 1 °C under a 12 h photophase and stained with lactophenol cotton blue: (**a**) ungerminated cyst [cy] and germinated cyst with a germ tube [gt] at 2 h; (**b**) germinated cyst with an elongating hypha [hy] at 8 h; (**c**) cyst bearing a long hypha [hy] at 24 h.

Representative microscopic observations were used to document the germination criterion applied in all media. Cysts incubated in MMY supplemented with 0.2% yeast extract showed ungerminated cysts and cysts bearing short germ tubes at 2 h (Figure 2a). At 8 h, growing hyphae with a conspicuous vacuole were observed (Figure 2b). At 24 h, cysts bearing long hyphae were present (Figure 2c).

### 3.2. Effect of Leptolegnia chapmanii on Aedes aegypti Larvae

**Figure 3:**
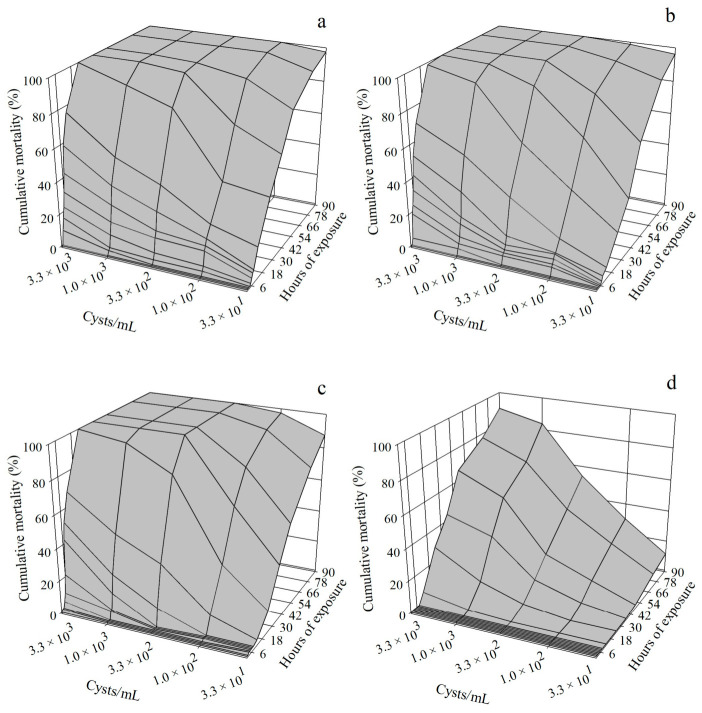
Relative cumulative mean mortality of *Aedes aegypti* first (**a**), second (**b**), third (**c**) and fourth (**d**) instar larvae after exposure to *Leptolegnia chapmanii* ARSEF 12829 cysts at different concentrations (3.3 × 10^1^–3.3 × 10^3^ cysts/mL) at 25 ± 1 °C and under a 12 h photophase for up to 90 h.

Cumulative mortality increased with cyst concentration and exposure time in all larval instars (Figure 3a–d; Table 1). At 3.3 × 10^3^ cysts/mL, final cumulative mortality at 90 h reached 100% in L1, L2 and L3 larvae, and 91.2 ± 8.7% in L4 larvae. At 3.3 × 10^1^ cysts/mL, the corresponding values were 96.9 ± 1.2%, 98.1 ± 1.2%, 88.7 ± 2.4% and 3.0 ± 1.6%, respectively. At 1.0 × 10^2^ cysts/mL, mortality remained high in L1–L3 larvae (95.9–98.4%) but was 17.3 ± 4.8% in L4 larvae. For each instar, the effect of cyst concentration on final mortality was significant (L1: F_4,27_ = 3.7; L2: F_4,27_ = 5.9; L3: F_4,27_ = 5.5; L4: F_4,27_ = 7.9; all *p* < 0.001; Table 1).

The shortest lethal times were recorded for L1 larvae at 3.3 × 10^3^ cysts/mL, with LT_50_ and LT_90_ values of 8.5 and 15.6 h, respectively (Table 1). In L4 larvae, LT values could not be calculated for 3.3 × 10^1^ or 1.0 × 10^2^ cysts/mL because mortality remained below 50%. At 3.3 × 10^2^ cysts/mL, the LT_50_ for L4 larvae was 85.5 h; the corresponding LT_90_ estimate exceeded the 90 h observation period and is therefore shown only in Table 1. At 1.0 × 10^3^ and 3.3 × 10^3^ cysts/mL, LT_50_ values for L4 larvae were 56.1 and 44.3 h, respectively.

Lethal concentration estimates also increased with larval instar (Table 2). At 12 h, LC_50_ values were 2.9 × 10^2^ cysts/mL for L1 larvae, 7.1 × 10^2^ cysts/mL for L2 larvae, and 9.3 × 10^2^ cysts/mL for L3 larvae. For L4 larvae, mortality at 12 h was insufficient for LC estimation; at 24 h, LC_50_ and LC_90_ values were 6.4 × 10^3^ and 3.6 × 10^4^ cysts/mL, respectively. Hyphae and/or zoosporangia were observed on or within all examined cadavers from treated groups, whereas control mortality remained low (≤6.5%).

### 3.3. Early Infection Stages in Third-Instar Larvae

**Figure 4:**
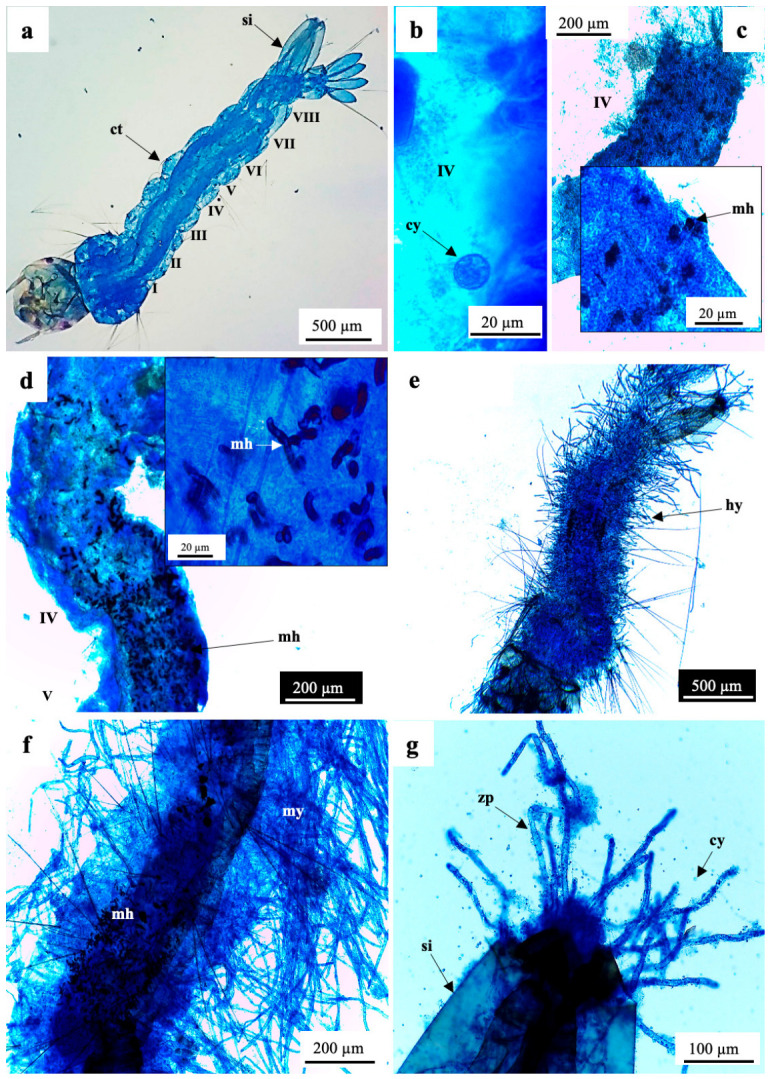
Development of *Leptolegnia chapmanii* ARSEF 12829 in third-instar *Aedes aegypti* larvae after exposure to cysts for up to 48 h at 25 ± 1 °C under a 12 h photophase. Specimens were stained with lactophenol cotton blue [33]: (**a**) uninfected L3 larva showing abdominal segments I–VIII, cuticle [ct], and siphon [si]; (**b**) ungerminated cysts [cy] adhered to the cuticle, 1 h after exposure; (**c**) dissected midgut showing melanized hyphal structures [mh] in abdominal segment IV 3 h after exposure; (**d**) aggregation of melanized hyphae [mh] in the midgut between abdominal segments IV and V 8 h after exposure; (**e**) hyphal development [hy] within the larval body and on its surface 24 h after exposure; (**f**) extensive mycelial growth [my] inside and on the surface of the larva 48 h after exposure; (**g**) hyphae [hy] around the siphon [si], with associated cyst-like propagules [cy] 48 h after exposure.

A representative uninfected third-instar larva is shown in Figure 4a as a reference, which shows the general body organization and abdominal segments. After exposure of L3 larvae to 1.0 × 10^3^ cysts/mL, a few cysts were observed attached to the cuticle at 30 min and 1 h; Figure 4b shows an ungerminated cyst on abdominal segment IV at 1 h. No external germ tubes were observed on attached cysts at these time points. At 3 h, melanized hyphal structures were observed in the dissected midgut (Figure 4c). At 8 h, melanized hyphae were visible in the midgut region corresponding to abdominal segments IV–V (Figure 4d). At 24 h, hyphae were observed within the larval body and on the surface (Figure 4e). At 48 h, abundant external mycelial growth was present on the larval surface, especially around the siphon and associated cyst-like propagules were observed (Figure 4f,g).

### 3.4. Histopathology of Infected L3 Larvae

**Figure 5:**
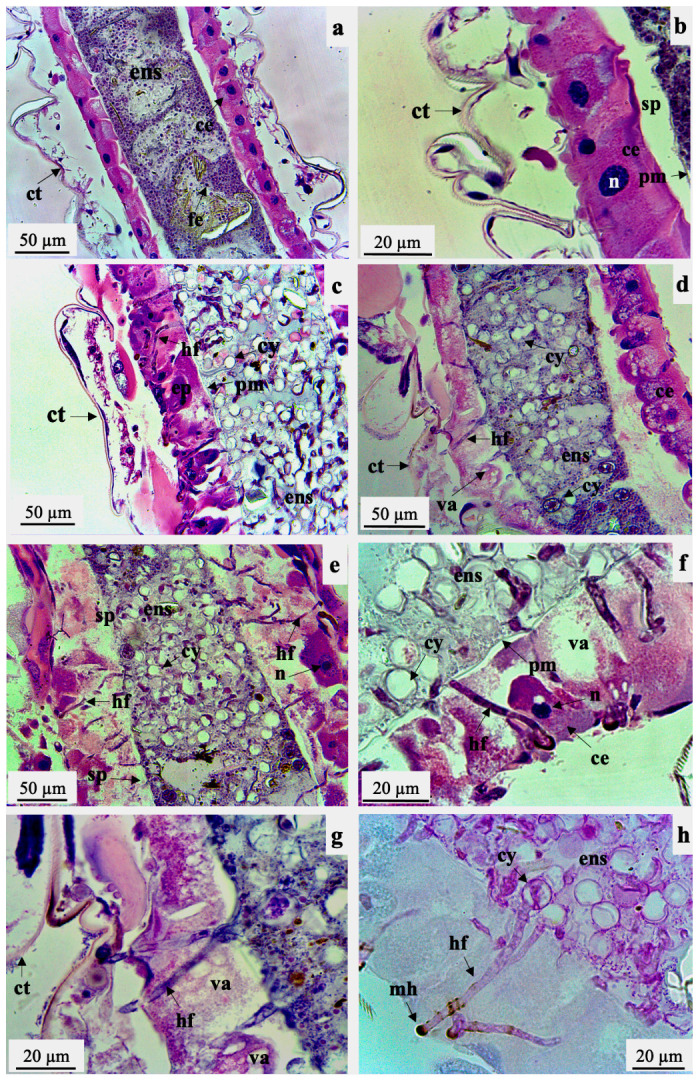
Longitudinal sections of third-instar *Aedes aegypti* larvae treated with water (controls, (**a**,**b**)) or exposed for 3 h to *Leptolegnia chapmanii* ARSEF 12829 cysts (1 × 10^3^ cysts/mL; (**c**–**h**)) at 25 ± 1 °C under a 12 h photophase. Sections were stained with hematoxylin–eosin (HE; (**a**–**g**)) or PAS (**h**). Control larvae (**a**,**b**) show preserved midgut organization, including epithelial cells [ce], nuclei [n], cuticle [ct], endoperitrophic space [ens], food elements [fe], peritrophic matrix [pm] and sub-peritrophic space [sp]. In infected larvae (**c**–**e**), hyphal filaments [hf] are observed in the endoperitrophic space [ens] and crossing the peritrophic matrix [pm] and epithelial layer [ep] toward the hemocoel; epithelial cells appear disorganized and disrupted, and the sub-peritrophic space [sp] remains evident but altered. Germinated and ungerminated cysts [cy] are present in the endoperitrophic space. Panels (**f**,**g**) show marked cytoplasmic vacuolization [va] and disruption of midgut epithelial cells [ce] associated with hyphal filaments [hf]. Panel (**h**), showing a section stained with PAS, shows PAS-positive cysts [cy] and hyphal filaments [hf], with melanized hyphae [mh] extending into the hemocoel.

In control L3 larvae, the median region of the mesenteron showed a single layer of cubic to cylindrical epithelial cells, a clearly delimited peritrophic matrix and a well-defined sub-peritrophic space (Figure 5a,b). After 3 h of exposure to 1.0 × 10^3^ cysts/mL, the midgut epithelium was disorganized and disrupted, and the sub-peritrophic space remained evident but appeared altered and irregular (Figure 5c–g). Small vacuoles were present in the apical region of some epithelial cells, and extensive cytoplasmic vacuolization was observed in many cells (Figure 5d,f,g).

Hyphae crossed the peritrophic matrix and the epithelial layer from the endoperitrophic space toward the hemocoel (Figure 5d,e). Germinated and ungerminated cysts were present in the endoperitrophic space (Figure 5e). PAS staining enhanced the visualization of cysts, hyphae and melanized hyphal segments in the hemocoel (Figure 5h). No oomycete structures were observed in or crossing the cuticle in sections obtained 3 h after exposure (Figure 5a–g).

## 4. Discussion

This study extends previous descriptions of *Leptolegnia chapmanii* infection by connecting three levels of evidence in the same Brazilian isolate: in vitro cyst germination in low-cost nutritional media, early in vivo invasion of the larval midgut and instar-dependent larvicidal activity. Earlier studies established the life cycle of *L. chapmanii* and demonstrated cuticular or oral infection routes in mosquito larvae [18,19,20]. In contrast, the present work shows that cysts of ARSEF 12829 can germinate rapidly under favorable nutritional conditions and that during the first hours after exposure, this capacity is temporally associated with hyphal penetration of the peritrophic matrix and midgut epithelium in *Aedes aegypti*. Thus, the main contribution is not the description of a new life cycle, but the integration of propagule activation, early gut invasion and larval susceptibility in a Brazilian isolate relevant to mosquito control.

The germination assays are also relevant from an applied perspective because they identify culture conditions that favor rapid cyst activation. Seed extracts supported high germination without requiring more elaborate culture media, whereas the minimal medium required yeast extract supplementation. This agrees with previous work showing that inexpensive plant-based substrates can support *L. chapmanii* or *L. giganteum* development and larvicidal activity [23,24,25]. However, because pH and osmotic conditions were not measured in the present assays, the observed pattern should be interpreted as a medium-dependent response rather than as evidence for the effect of a single nutritional factor. Plant-derived lipids or sterol-related compounds may have contributed to this response [26], but this possibility requires targeted biochemical evaluation.

The infection sequence documented here places the digestive tract at the center of the early phase of pathogenesis. A few cysts were seen attached to the cuticle during the first 30–60 min, and no histological evidence of cuticular penetration was detected at 3 h. In contrast, dissected larvae already contained melanized hyphal structures in the midgut by 3 h, and histological sections showed ungerminated and germinated cysts in the en-doperitrophic space, with hyphae crossing the peritrophic matrix and epithelium into the hemocoel. This supports oral entry as the predominant early pathway under the present conditions, while not excluding cuticular entry or additional infection events at later times. The epithelial disorganization and cytoplasmic vacuolization observed at this stage indicate that tissue damage begins soon after midgut invasion.

The marked difference among larval instars indicates that host developmental stage strongly influences infection success. Mortality remained high in L1–L3 larvae, even at relatively low cyst concentrations, whereas L4 larvae required greater exposure and responded more slowly. Earlier reports on mosquito pathogens have also described lower susceptibility in advanced larval stages, which may reflect lower ingestion before pupation, a shorter remaining larval period and stronger mechanical or physicochemical barriers in the gut, including the peritrophic matrix [13,17,37,38,39]. Because larvae were fasted before exposure, absolute mortality values should be extrapolated cautiously to field conditions, where feeding status and gut content vary.

From a biological control perspective, the results point to two linked priorities: producing cysts with rapid activation capacity and targeting larval populations in which early instars predominate. Under field or container conditions, infection success will also depend on zoospore-host encounter rates, larval density, water surface area and fluctuating environmental conditions [21,40]. Previous work showed that short-term temperature stress can reduce *L. chapmanii* activity and affect the production of infective structures on cadavers, while compatibility with diflubenzuron and neem oil suggests that this oomycete may be integrated with other larval control agents [21,22]. The present data add that rapid cyst activation and early midgut invasion should be considered when evaluating propagule quality for biological control.

Because ARSEF 12829 was originally isolated from *A. aegypti* in Brazil [12], these findings highlight the value of studying Brazilian isolates obtained from mosquito breeding sites. Future work should evaluate whether media that improve cyst activation also enhance persistence, recycling on cadavers and larvicidal activity under semi-field or field conditions, especially in containers with different organic matter contents and larval densities.

Taken together, these results show that the pathogenic activity of *L. chapmanii* ARSEF 12829 depends on the integration of propagule activation, early midgut invasion and host developmental stage. By combining germination assays, histopathology and mortality data, this study refines the current understanding of early *L. chapmanii* pathogenesis and identifies biologically relevant traits that may guide propagule production and future biological control applications.

## Figures and Tables

**Table 1:** Cumulative mean mortality ± SE (standard error) at 90 h and lethal time estimates (LT_50_ and LT_90_, h), with 95% confidence intervals (95% CIs) and slope ± SE, for *Aedes aegypti* larval instars (L1–L4) after exposure to *Leptolegnia chapmanii* ARSEF 12829 cysts (3.3 × 10^1^–3.3 × 10^3^ cysts/mL) or water only (control) at 25 ± 1 °C under a 12 h photophase.

Instar	Cysts/mL	Mortality ± SE	LT_50_ (95% CI)	LT_90_ (95% CI)	Slope ± SE
L1 *	3.3 × 10^1^	96.9 ± 1.2b	34.2 (15.2–52.1)b–e	71.9 (53.7–108.7)de	0.03 ± 0.003
	1.0 × 10^2^	95.9 ± 2ab	26.1 (7.6–44.9)a–e	49.1 (34.1–53.4)bc	0.06 ± 0.005
	3.3 × 10^2^	100ab	15.4 (1.4–28.3)a–d	30.1 (19.9–65.3)a–e	0.09 ± 0.008
	1.0 × 10^3^	100a	12.7 (5.4–20.4)a–c	23.7 (17.2–42.5)ab	0.11 ± 0.011
	3.3 × 10^3^	100a	8.5 (5.2–12.8)a	15.6 (11.9–25.2)a	0.18 ± 0.021
L2 *	3.3 × 10^1^	98.1 ± 1.2c	45.6 (33–58.3)e	78.1 (64.4–101.4)e	0.04 ± 0.003
	1.0 × 10^2^	98.4 ± 1.2bc	31.2 (15.9–45.8)b–e	63.8 (48.7–92.7)c–e	0.04 ± 0.004
	3.3 × 10^2^	100ab	20.1 (12.2–28.1)a–d	37.8 (29.5–54.7)b–d	0.07 ± 0.007
	1.0 × 10^3^	100ab	12.6 (2.2–22.5)a–d	25.1 (16.9–51.1)a–c	0.01 ± 0.009
	3.3 × 10^3^	100a	9.5 (2.9–16.4)ab	18.1 (12.6–37.5)ab	0.14 ± 0.015
L3 *	3.3 × 10^1^	88.7 ± 2.4c	46.5 (41.9–51.3)de	72.2 (66.2–80)e	0.05 ± 0.004
	1.0 × 10^2^	98.4 ± 1.2bc	33.5 (19.6–47.1)c–e	62.9 (48.9–89.6)c–e	0.04 ± 0.004
	3.3 × 10^2^	100ab	14.8 (2.1–26.3)a–d	23.3 (16.5–67.6)a–e	0.15 ± 0.014
	1.0 × 10^3^	100ab	12.1 (4.8–20.7)a–c	20.1 (14.2–45.7)ab	0.02 ± 0.016
	3.3 × 10^3^	100a	9.6 (3.3–17.1)ab	15.1 (10.7–44.8)ab	0.18 ± 0.021
L4 *	3.3 × 10^1^	3 ± 1.6c	**	**	**
	1.0 × 10^2^	17.3 ± 4.8c	**	**	**
	3.3 × 10^2^	57.3 ± 11.7bc	85.5 (74.9–98.5)f	140.9 (123–169)f ***	0.02 ± 0.003
	1.0 × 10^3^	82 ± 6.2ab	56.1 (33.6–79.5)ef	106.5 (82.2–161)ef ***	0.03 ± 0.003
	3.3 × 10^3^	91.2 ± 8.7a	44.3 (21.5–65.9)de	89.7 (67.7–138)ef	0.03 ± 0.003

Values of mortality for the same larval instar followed by different letters (a–c) were significantly different based on ANOVA and SNK test. * For each larval instar, cumulative mortality differed significantly among cyst concentrations (L1: F_4,27_ = 3.7; L2: F_4,27_ = 5.9; L3: F_4,27_ = 5.5; L4: F_4,27_ = 7.9; all *p* < 0.001). ** Mortality insufficient (<50%) to calculate lethal times; values of LT_50_ or LT_90_ obtained for different concentrations and all instars in the same column followed by different letters (a–f) were significantly different based on their CI; control mortalities were ≤5.8%. *** LT_90_ estimate exceeded the 90 h observation period and should therefore be interpreted as an extrapolated value.

**Table 2:** Lethal concentration of *Leptolegnia chapmanii* (cysts/mL) required to kill 50% and 90% (LC_50_ and LC_90_) of *Aedes aegypti* larvae (L1–L4), with their respective confidence intervals (CIs) and slope ± SE, after 12 h (L1–L3) or 24 h (L4) of exposure to cysts (3.3 × 10^1^–3.3 × 10^3^ cysts/mL) at 25 ± 1 °C and under 12 h photophase.

Instar	Hour	LC_50_ (95% CI)	LC_90_ (95% CI)	Slope ± SE
L1 *	12	2.9 × 10^2^ (2.1 × 10^2^–4.1 × 10^2^)a	6.6 × 10^3^ (3.7 × 10^3^–1.5 × 10^4^)a	1.0 ± 0.1
L2 *		7.1 × 10^2^ (5.2 × 10^2^–1.0 × 10^3^)b	1.1 × 10^4^ (5.9 × 10^3^–2.5 × 10^4^)a	1.1 ± 0.1
L3 *		9.3 × 10^2^ (7.1 × 10^2^–1.3 × 10^3^)b	1.1 × 10^4^ (6.0 × 10^3^–2.2 × 10^4^)a	1.2 ± 0.1
L4 **	24	6.4 × 10^3^ (4.3 × 10^2^–1.1 × 10^5^)	3.6 × 10^4^ (1.8 × 10^4^–1.1 × 10^5^)	1.7 ± 0.2

Values of LC_50_ or LC_90_ obtained for the same exposure time followed by different letters (a,b) were significantly different based on their CI; * mortality at 24 h was too high; ** at 12 h was insufficient to calculate LC; no control mortality observed at 24 h.

## Data Availability

All data generated or analyzed during this study are included in this published article. Additional raw data are available from the corresponding author upon reasonable request.
